# Impact of Socioeconomic Deprivation on Access to Diabetes Technology in Children With Type 1 Diabetes Mellitus

**DOI:** 10.7759/cureus.86804

**Published:** 2025-06-26

**Authors:** Haris Shoaib, Shahida Ahmed, Ruth Francks, Sulaiman Hussain, Haider A Chaudhary, Shiza Shoaib, Sanjay Rawal

**Affiliations:** 1 Trauma and Orthopaedics, Royal Preston Hospital, Preston, GBR; 2 Paediatrics, Basildon University Hospital, Basildon, GBR; 3 Trauma and Orthopaedics, Royal Blackburn Teaching Hospital, Blackburn, GBR; 4 Surgery, Cumberland Infirmary, Carlisle, GBR; 5 Medical Education, GKT (Guy's, King's and St. Thomas's) School of Medicine, King’s College London, London, GBR

**Keywords:** diabetes technology, insulin pumps, paediatric endocrinology, socio-economic impact, type 1 diabetes mellitus (t1d)

## Abstract

Aims

The aim of this study was to assess the impact of socioeconomic deprivation on access to diabetes technology, specifically insulin pumps, and its outcome in children with type 1 diabetes.

Methods

This was a retrospective, observational, single-centre study of patients attending the paediatric unit at Basildon University Hospital, Basildon, United Kingdom. The study included all patients actively receiving diabetic care as of April 2023, including those with access to insulin pumps between January 2012 and April 2023, with their HbA1c values assessed before and after initiating insulin pump treatment. Deprivation quintiles were calculated. Statistical significance was calculated via chi-square tests, one-way ANOVA, and logistic regression approximation analysis.

Results

Included in the study were 243 children and young people (CYP) with a mean age of 13 years, 117 of whom were male (48%). Of this caseload, 48 had active access to insulin pumps with a mean deprivation quintile of 3.15 (SD 1.44). Quintile 1 identified the most deprived populations, and quintile 5, the least deprived. The insulin pumps were most accessible for CYP in the least deprived quintile compared to those in the most deprived quintile (31% vs. 10%; p<0.01). Within the caseload, following initiation of treatment, CYP in the most deprived quintile had the highest mean HbA1c values compared to the lowest values in the least deprived quintile (67.83 (SD 24.72) vs. 51.64 (SD 9.45); p=0.027). HbA1c outcomes were available for 35 CYP using pumps, with no statistically significant link to deprivation (p=0.348). CYP of White ethnicity had the highest use of insulin pumps compared to any other ethnicity (88% vs. 12%, p<0.0001).

Conclusions

Inequalities in access to diabetic technology still exist, with CYP in the least deprived quintile and those of White ethnicity experiencing greater access to technology. CYP from all deprivation quintiles experienced positive glycaemic control with technology use, suggesting improving access to technology may reduce glycaemic disparities in deprived populations. The most deprived populations might be disadvantaged due to their lack of exposure and awareness about newer technologies and advancements in diabetes care, as well as sub-optimal engagement with diabetes services, which is often seen in these cases. Further research is yet required to address these health inequalities.

## Introduction

Over the past 50 years, diabetes management has evolved significantly, with growing emphasis on supporting self-management to optimise glycaemic control. This shift has been largely driven by advancements in technology, leading to the development of a range of devices, from glucose monitoring to insulin delivery devices and hybrid systems that combine both functions. 

Insulin pumps have been used in the management of diabetes since the 1970s, when they were mainly exclusive to those from higher socioeconomic backgrounds [1]. Following many years of innovation and development, insulin pumps are now more commonly used within the United Kingdom (UK) paediatric population, with use of these pumps increasing by 10% from 2022/2023 to 2023/2024 [2]. The use of insulin pumps has been shown to positively affect glycaemic control, particularly in these younger patient groups [3]. 

Despite robust evidence to support the use of diabetic technology in the management of type 1 diabetes (T1DM), there are still widespread discrepancies in access to these technologies [4]. The widening of the socioeconomic gradient in the UK over the last decade has had a significant role in increasing health inequalities. These inequalities may contribute to high-risk lifestyles such as smoking, poor diet, and reduced physical activity, negatively impacting the management of type 1 diabetes in children and young people (CYP) [5]. The UK poverty report found that 3.4 million households have reported a lack of funds for food, with 4.2 million children (approximately three in 10) living in circumstances below UK poverty levels [6]. The need for close monitoring in the management of T1DM can also negatively affect the financial and occupational situations of families [7]. Accessibility and experiences of these technologies are limited by education and awareness, such as requiring the ability to use multiple platforms for data analysis to optimise care, where interpretation of numerical data remains a limiting factor [8,9]. 

The UK National Paediatric Diabetes Audit (NPDA) suggests that the incidence of T1DM in CYP aged less than or equal to 18 in 2020/21 in England and Wales was approximately 29,892 [10]. The more recent statistics from the annual report in 2023/24 data suggest there are 35,122 cases of T1DM in those aged 18 and younger, which shows a 17% increase compared to the reported figures in the NPDA 2020/21 [2,10]. There is a lack of clear data investigating whether T1DM has increased at a greater rate in more deprived areas. 

According to the NPDA 2023/24, the average HbA1c gap remains similar between the most and least deprived quintiles in the UK paediatric population: 66.0 and 60.0 mmol/mol, respectively, in 2023/24, compared to 66.8 and 59.9 mmol/mol in 2022/23 [2]. This has also been closely linked to ethnic groups, and the statistics show a significant disparity in average HbA1c values in Black versus White ethnicity equivalents, 70.8 versus 63.1 mmol/mol, respectively [2]. Consequently, patients from lower socioeconomic backgrounds are more likely to visit the emergency department regarding diabetes-related health problems [11]. Given the increase in the number of CYP living with T1DM and the differences shown in glycaemic control, this may suggest that disparity exists between the use of insulin technologies for the management of T1DM between different socioeconomic groups. This is evident from the latest NPDA report, which shows that technology use is less prevalent among CYP living in more deprived areas and ethnic minorities [2]. 

The NPDA 2023/24 suggests that 55% of CYP received treatment via insulin pumps in the management of T1DM [2]. The use of continuous subcutaneous insulin infusion pumps has been beneficial for patients with raised HbA1C levels and has been shown to reduce hypoglycaemia frequency [12]. Insulin pumps are also effective in being able to deliver increments as small as 0.025 units, which allows for further precision in insulin dosing in comparison to existing insulin pens or syringes [13]. The insulin pumps have an added benefit of adapting to a child’s lifestyle, which can often be unpredictable in meal intake and activity, aiding parents or guardians in managing the condition, particularly when children are unable to communicate their symptoms [3,14]. 

The NPDA 2023/24 reported a lower percentage of insulin pump use among CYP in the most deprived quintile (50%) compared to the least deprived quintile (59.5%) within England and Wales [2]. Children from higher socioeconomic backgrounds have shown increased pump use, which may be attributed to higher levels of parental education, understanding, and acceptability of pump use [4]. Insulin pump users with parents or guardians from higher socioeconomic backgrounds may experience greater collaboration with healthcare providers to promote optimal diabetes management, thus promoting use of this technology within their own communities [15]. 

There exists limited literature linking socioeconomic deprivation and access to diabetes technologies in CYP living with diabetes within the UK, highlighting the need for further well-powered research in this field [16]. The management of T1DM in the UK varies depending upon local hospital policies, affecting the levels of access to the technologies discussed above, and offering different levels of funding depending upon location [17]. This study aims to assess the relationship between socioeconomic deprivation status and its effect on access to technology in the management of CYP with T1DM. Secondary objectives include exploring associated glycaemic outcomes and identifying potential systemic barriers to care. 

## Materials and methods

This was a retrospective, observational, single-centre study of CYP with T1DM receiving ongoing management by the diabetes services at Basildon University Hospital, Basildon, UK, within the Mid and South Essex NHS Foundation Trust, as of April 2023. The Mid and South Essex Sustainability and Transformation Partnership delivers integrated diabetic care to a diverse population with multifarious needs [18]. Access to insulin pumps for patients living within catchment areas of local clinical commissioning groups was in line with the Mid and South Essex NHS Foundation Trust commissioning recommendations [19] and NHS England national guidance [20].

Study population

All patients attending diabetes services were identified by the diabetes clinical nurse specialists at Basildon University Hospital, including individuals with active use of insulin pumps and their timelines of access to technology. Formal NHS Research Ethics Committee review was not required, in accordance with the Health Research Authority (HRA) guidance [21]. Data were collected and anonymised in compliance with NHS data governance protocols and local Caldicott guardian oversight. All CYP aged ≤19 years with a clinical diagnosis of T1DM, actively receiving ongoing care from the paediatric diabetes team at Basildon University Hospital as of April 2023, were considered eligible. Individuals were included if they had a valid postcode (required for calculation of socioeconomic deprivation quintile) and at least one recorded HbA1c measurement in the electronic medical record. Patients without a postcode or with insufficient clinical records to calculate socioeconomic deprivation quintiles were excluded. The study cohort included patients managed between January 2012 and April 2023.

Data collection

NHS hospital numbers of all patients included in the study were cross-referenced with electronic health records held on the Mid and South Essex NHS Foundation Trust (Acute Care Portal) and Basildon University Hospital electronic patient records (Medway EPR; System C Healthcare Ltd, Kent, UK). Acute Care Portal allows data to be uploaded by health administrators and clinicians, including medical notes, pathology and radiology results, and documentation such as discharge letters and clinic letters. Data from all three hospitals within the Mid and South Essex NHS Foundation Trust can be accessed using this software. Both Acute Care Portal and Medway EPR are part of the NHS Spine [22], which stores patient demographic data, such as self-reported ethnicities and postcodes.

Measures of deprivation

The level of socioeconomic deprivation was assessed using the English Indices of Deprivation 2019 [23]. Deprivation deciles are based on the Index of Multiple Deprivation 2019 (IMD 2019).

Each patient’s residential postcode was linked to a Lower Layer Super Output Area (LSOA), which was then mapped to an IMD decile. The most deprived 10% of populations in England are represented by decile 1, with the least deprived 10% of populations represented by decile 10. In order to represent data in accordance with the National Diabetes Audit, the deprivation data were divided into deprivation quintiles, with quintile 1 representing the most deprived 20% of English populations, and quintile 5 representing the least deprived 20% of populations.

Statistical analysis

Baseline characteristics are presented as mean±SD (standard deviation) for continuous data, and as number with percentage of participants for any discrete variables.

To assess statistical significance across all deprivation quintiles for categorical variables (quintile populations, sex, ethnicity, and technology use), chi-squared tests (χ2) were used. For the evaluation of statistical significance across quintiles with continuous data, ordinary one-way ANOVA tests were performed.

For use of diabetes technology, differences in HbA1c were obtained pre-initiation of insulin pump treatment (>6 months from July 2023) and post-initiation (<6 months from July 2023). Differences in HbA1c were evaluated using one-way ANOVA tests, with p-values < 0.05 set as the threshold for statistical significance. To assess differences in HbA1c within the patient caseload, including those not using insulin pumps, only individuals with recorded HbA1c values >6 months and <6 months from July 2023 were included.

Logistic regression approximation analysis was also performed with the available data to assess predictors of insulin pump usage and quantify how deprivation level affects the odds of insulin pump usage whilst adjusting for confounders. The dependent variable was current insulin pump use (evaluated by yes/no), and the independent variables included IMD deprivation quintile, age, sex, and ethnicity. Odds ratios (ORs) with 95% confidence intervals (CIs) were calculated, with model fit and multicollinearity assessed prior to final analysis.

All statistical analyses were performed using GraphPad Prism version 10.0.2 (Dotmatics, Boston, Massachusetts, United States) [24].

## Results

Of the 243 CYP with a diagnosis of T1DM actively receiving diabetes care at Basildon University Hospital, 16 were excluded from the study due to insufficient clinical records and missing post-codes for the calculation of deprivation quintiles. Thus, a total of 227 CYP with T1DM were included in this study. Baseline demographic data of the study population, stratified by IMD 2019 quintiles, are shown in Table 1.

**Table 1:** Baseline demographic data of patient caseload stratified by Indices of Multiple Deprivation 2019* quintiles (N=227) *Indices of Multiple Deprivation 2019 [23]

Characteristics	Total	Quintile 1	Quintile 2	Quintile 3	Quintile 4	Quintile 5	p-value
Number of patients, n (%)	227 (100%)	48 (21.1%)	62 (27.3%)	42 (18.5%)	21 (9.3%)	54 (23.8%)	<0.001
Age (years), mean ± SD	13 ± 4	13 ± 4	12 ± 4	11 ± 4	13 ± 4	12 ± 4	0.347
Sex, n (%)							0.74
Male	111 (48.9%)	23 (47.9%)	41 (66.1%)	18 (42.9%)	7 (33.3%)	22 (40.7%)	<0.001
Female	116 (51.1%)	25 (52.1%)	21 (33.9%)	24 (57.1%)	14 (66.7%)	32 (59.3%)	0.118
Technology, n (%)							<0.001
Insulin pump use	48 (21.1%)	5 (10.4%)	16 (25.8%)	9 (21.4%)	3 (14.3%)	15 (27.8%)	0.007
No insulin pump use	179 (78.9%)	43 (89.6%)	46 (74.2%)	33 (78.6%)	18 (85.7%)	39 (72.2%)	0.008

Analysis indicated that the distribution across socioeconomic quintiles based on the Indices of Multiple Deprivation 2019 was significantly uneven (p<0.001), with the highest representation from Quintile 2 (27.3%) and the lowest from Quintile 4 (9.3%). The mean age of the population was 13 ± 4 years, with no significant age difference observed across all five quintiles (p=0.347). Sex distribution was largely similar across deprivation levels (p=0.740). Within the caseload, 48 patients (21.1%) were actively using insulin pumps as part of their diabetes care.

Regarding the use of insulin pump therapy, significant variability by socioeconomic status was indicated (p=0.007), with the lowest uptake in the most deprived quintile (Quintile 1, 10.4%) and the highest in Quintile 5 (27.8%) (Tables 1, 2). For those patients using insulin pump therapy (n=48), a significant disparity in ethnic group distribution is noted, with 87.5% of those patients identifying as White (p<0.001). The number of Quintile 1 patients was markedly lower in active insulin pump use (10.4%) compared to Quintile 5 (31.3%; p=0.007).

**Table 2:** Baseline demographic data of patients actively using insulin pumps, stratified by Indices of Multiple Deprivation 2019* quintiles (N=48) *Indices of Multiple Deprivation 2019 [23]

Characteristics	Total	Quintile 1	Quintile 2	Quintile 3	Quintile 4	Quintile 5	p-value
Number of Patients, n (%)	48 (100%)	5 (10.4%)	16 (33.3%)	9 (18.8%)	3 (6.3%)	15 (31.3%)	0.007
Sex							0.773
Male	23 (47.9%)	3 (60%)	9 (56.3%)	3 (33.3%)	1 (33.3%)	7 (46.7%)	0.052
Female	25 (52.1%)	2 (40%)	7 (43.8%)	6 (66.7%)	2 (66.7%)	8 (53.3%)	0.171
Ethnicity							<0.001
White (British, any other White background)	42 (87.5%)	3 (60%)	15 (93.8%)	8 (88.9%)	3 (100%)	13 (86.7%)	0.005
Mixed (White and Black African)	1 (2.1%)	0 (0%)	1 (6.3%)	0 (0%)	0 (0%)	0 (0%)	0.406
Mixed (any other background)	1 (2.1%)	1 (20%)	0 (0%)	0 (0%)	0 (0%)	0 (0%)	0.406
Not stated	4 (8.3%)	1 (20%)	0 (0%)	1 (11.1%)	0 (0%)	2 (13.3%)	0.478

HbA1c-related outcomes were available for 146 patients within the caseload (64.3%). Regarding glycaemic control, further statistically significant disparity was indicated between quintiles 5 and 1 (51.63 ± 9.45 mmol/mol and 7.84 ± 23.56 mmol/mol, respectively; p=0.008) (Tables 3, 4). Glycaemic control was observed to have improved on the introduction of insulin pump use, with mean HbA1c decreasing from 53.69 ± 13.61 mmol/mol at >6 months to 49.71 ± 8.47 mmol/mol at <6 months post initiation.

**Table 3:** HbA1c-related outcomes across Indices of Multiple Deprivation* quintiles for patients within the caseload (N=146) *Indices of Multiple Deprivation 2019 [23]

HBA1c	Total	Quintile 1	Quintile 2	Quintile 3	Quintile 4	Quintile 5	p-value
Number of patients, n (%)	146 (100%)	31 (21.2%)	41 (28.1%)	29 (19.9%)	12 (8.2%)	33 (22.6%)	0.004
HbA1c > 6 months, mean ± SD	67.73 ± 25.98	70.32 ± 24.72	63.07 ± 23.52	67.97 ± 26.35	75.17 ± 30.67	68.15 ± 28.40	0.629
HbA1c < 6 months, mean ± SD	59.58 ± 18.86	67.84 ± 23.56	58.15 ± 17.67	59.10 ± 12.37	66.17 ± 30.84	51.63 ± 9.45	0.008

**Table 4:** HbA1c-related outcomes for patients actively using insulin pump technology (N=35)

HBA1c	Total	Quintile 1	Quintile 2	Quintile 3	Quintile 4	Quintile 5	p-value
Number of patients, n (%)	35 (100%)	4 (11.4%)	11 (31.4%)	7 (20%)	1 (2.9%)	12 (34.3%)	0.015
HbA1c > 6 months, mean ± SD	53.69 ± 13.61	65.25 ± 32.81	51.45 ± 8.64	52.29 ± 7.09	50.00 ± 0.00	53.00 ± 11.16	-
HbA1c < 6 months, mean ± SD	49.71 ± 8.47	55.00 ± 17.32	50.00 ± 8.25	50.86 ± 5.05	44.00 ± 0.00	46.58 ± 5.82	-

**Figure 1:**
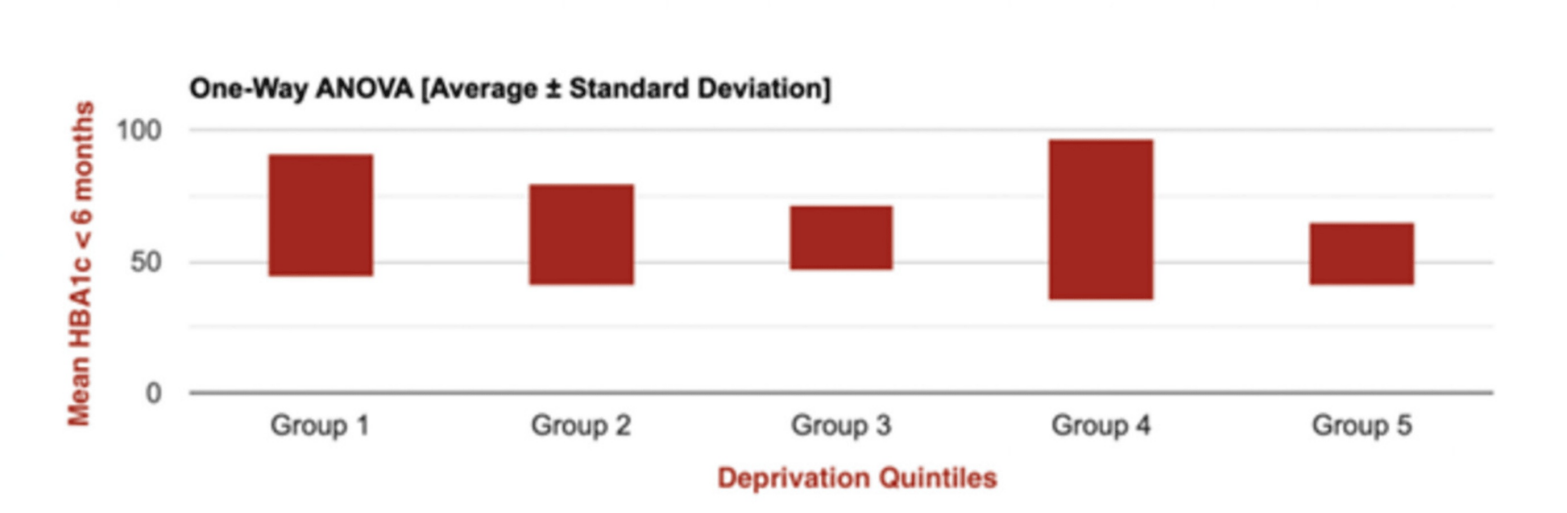
One-way ANOVA graph representing mean pre-insulin pump initiation HbA1c values for all patients within the caseload, stratified by IMD 2019* quintiles (N=146) *Indices of Multiple Deprivation 2019 [23]

To assess mean pre-insulin pump initiation HbA1c values for the entirety of the sample population with HbA1c-related data available (measured six months prior to July 2023) across all quintiles (n=146; Figure 1), a one-way ANOVA test was performed. There was no statistical significance in the difference observed (F(4, 140) = 0.5672; p = 0.6869). This highlights the potential that HbA1c value patterns are dependent on alternative factors in conjunction with deprivation levels.

**Figure 2:**
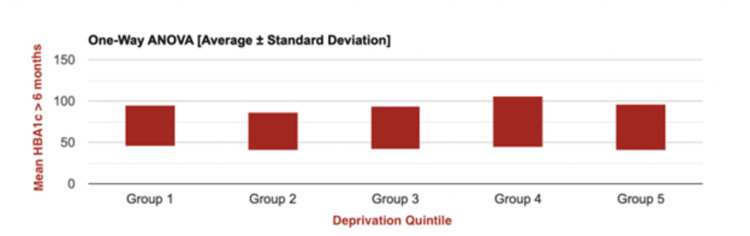
One-way ANOVA graph representing mean post-insulin pump initiation HbA1c values for all patients within the caseload, stratified by IMD 2019* quintiles (N=146) *Indices of Multiple Deprivation 2019 [23]

A further one-way ANOVA was carried out to assess mean post-insulin pump initiation HbA1c levels (measured within six months of July 2023) across all five quintiles for the entirety of the sample population with HbA1c-related data available (n=146) (Figure 2). This demonstrated a statistically significant difference in HbA1c across all five quintiles (F(4, 145) = 2.825; p=0.0271). This analysis indicated that those patients in the lowest quintiles had the highest mean HbA1c, compared with those in the highest quintiles, which had the lowest, a statistically significant difference.

**Figure 3:**
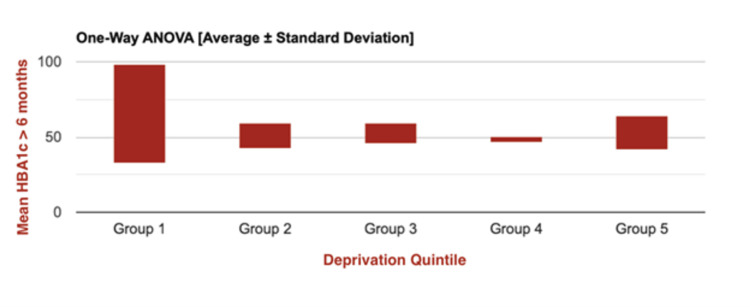
One-way ANOVA graph representing mean pre-insulin pump initiation HbA1c values for all patients with active use of insulin pumps, stratified by IMD 2019* quintiles (n=35) *Indices of Multiple Deprivation 2019 [23]

In order to determine the presence of statistically significant differences in mean HbA1c levels (measured within six months pre-insulin pump initiation) across the five deprivation quintiles amongst those patients actively using pump therapy (n=35), a further one-way ANOVA test was performed (Figure 3). No statistically significant differences were identified across all five quintiles (F(4,32) = 0.93; p = 0.457), suggesting no discernible link between deprivation quintile and baseline glycaemic control prior to initiation of insulin pump use.

**Figure 4:**
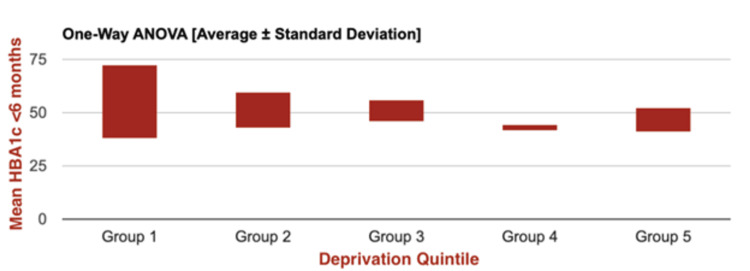
One-way ANOVA graph representing mean post-insulin pump initiation HbA1c values for all patients with active use of insulin pumps, stratified by IMD 2019* quintiles (n=35) *Indices of Multiple Deprivation 2019 [23]

A final one-way ANOVA test was performed to assess differences in mean HbA1c levels (measured within six months post-insulin pump initiation) across the five deprivation quintiles amongst those patients actively using pump therapy (n=35) (Figure 4). No statistically significant difference in mean HbA1c values across the quintiles was demonstrated (F(4, 31) = 1.1587; p=0.3479). Relatively consistent HbA1c levels across quintiles were also demonstrated, with a slight decline in the higher quintiles. These differences, however, were not statistically significant.

**Figure 5:**
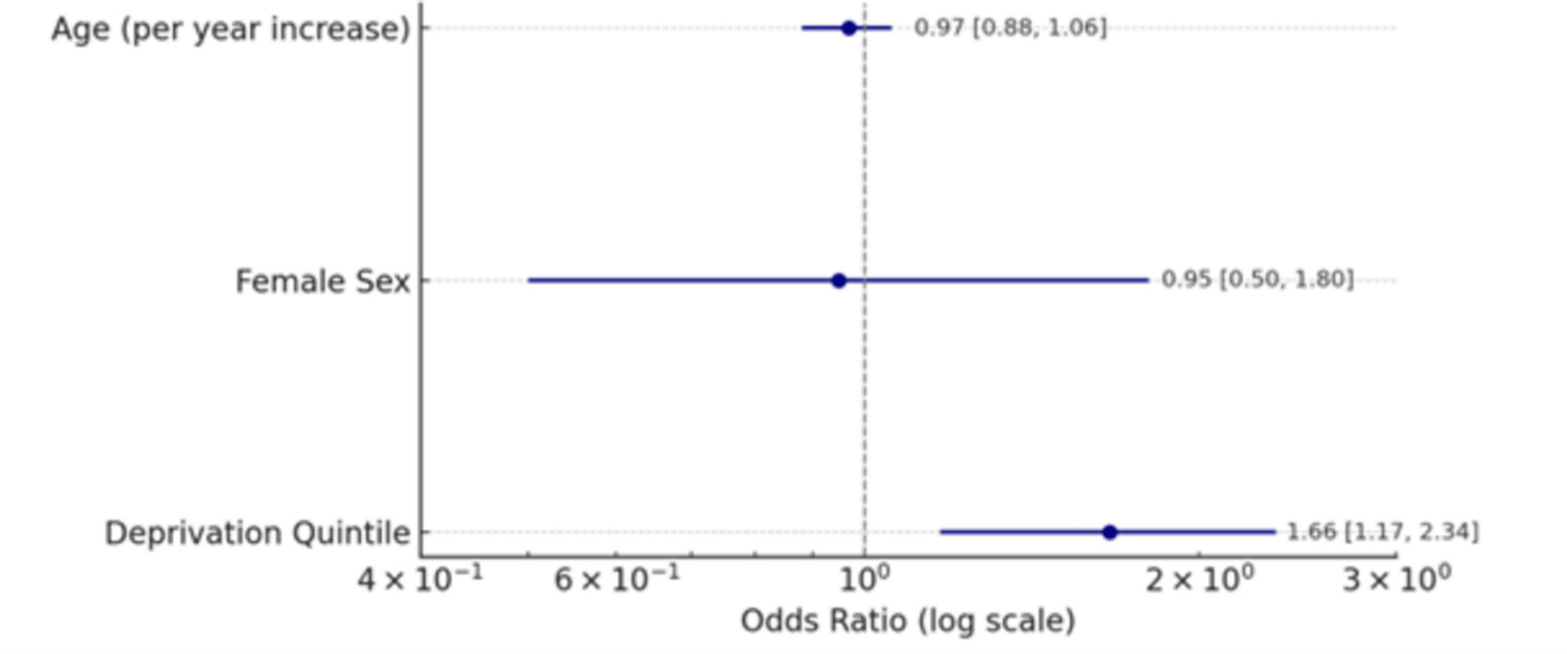
Odds ratio for insulin pump use of all patients within the caseload (N=227)

Logistic regression approximation of insulin pump use was performed on all patients within the caseload (n=227). This indicated that an increase in one unit of deprivation elicited a 66% increase in odds of pump use (OR 1.66, 95%CI 1.17-2.34; p=0.005), thereby showing that those patients from less deprived backgrounds were significantly more likely to use insulin pump technology (Figure 5).

## Discussion

In this study, we have shown that socioeconomic deprivation has a significant impact on access to diabetes technology, specifically insulin-delivery pumps. This is shown by the logistic regression analysis indicating that for each increase in deprivation incrementation, the odds of insulin pump usage increased by 66% (OR 1.66, 95% CI 1.17-2.34; p = 0.005), thus highlighting the vast healthcare inequality in the included cohort. This could be due to a number of reasons, such as poor healthcare literacy or navigation, or a possible lack of advocacy for these more deprived groups. Notably, as this technology is novel, it is likely that there is a lag in patient understanding and sustained engagement with the use of said technology in the more deprived groups, suggesting the need for the improvement of digital literacy education to the relevant groups [25]. This is demonstrated by the DESMOND (Diabetes Education and Self Management for Ongoing and Newly Diagnosed) program, which was set up in order to improve patient education regarding diabetes management, targeting those patients with low literacy rates [26]. This program subsequently elicited significant improvements in patient engagement with their diabetes care as well as understanding of the pathology itself, highlighting the importance of targeted patient education schemes in order to improve overall care of chronic conditions.

A study by Mehta et al. reported significant HbA1c improvements in patients transitioning to insulin pumps [27]. Regarding the impact of insulin delivery pumps on overall glycaemic outcomes in this study, the improvement in HbA1c control (from 53.69 to 49.71 mmol/mol) was not deemed to be statistically significant (p=0.1465). This suggests that access to and use of insulin delivery pumps are not to be relied upon in order to improve glycaemic control in a population of patients with T1DM; however, it should be noted that due to the limited sample size included in the study, this study did not reach the required power to be definitive [28]. A post-hoc analysis performed using G*Power 3.1 (Heinrich Heine University, Düsseldorf, Germany) suggests that approximately 80 patients would be required to detect a statistically significant difference of this magnitude with 80% power (α = 0.05). However, data from the NPDA 2023/24 suggest that for CYP within England and Wales, insulin pump use (median HbA1c 58.0 mmol/mol) improves glycaemic outcomes when compared to treatment with the use of multiple daily injections (median HbA1c 64.0 mmol/mol) [2]. As such, it is indicated that insulin pump use in conjunction with traditional methods such as patient education, psychosocial support, and multidisciplinary input is required to elicit a statistically significant improvement in glycaemic control [29,30].

The data collected in this study indicate a widespread need for access to insulin pumps across all quintiles, with 27 patients within the caseload awaiting pump initiation. This highlights that whilst clinicians are looking favourably at insulin delivery pumps as a therapeutic intervention for patients, there is either a lack of funding, lack of access to technology by the NHS Trust as a whole, lack of resources in diabetes services to meet demand in a timely manner, or potential delays in the referral process [31]. As such, there remains a need for logistical optimisation to ensure that all patients across all quintiles get access to the technology as soon as it is clinically indicated by the responsible clinician [28]. A practical example for how a structured pathway can have an impact on access to healthcare technology is exhibited by an initiative that took place at Birmingham Women's and Children's NHS Foundation Trust, where implementation of a dedicated pump co-ordinator role, structured patient education, and targeted multidisciplinary team meetings led to a substantial increase in insulin pump uptake and increased accessibility for all socio-economic groups [32]. 

In order to address the healthcare inequalities identified, different approaches need to be adopted to meet the specific needs of the locality, considering ethnic backgrounds and language barriers. It has been shown that culturally adapted patient education programs can improve engagement in these areas of social deprivation [33]. Other methods of targeting local communities include multilingual information leaflets, the use of interpreters at health education events, and the organisation and delivery of smaller group health education sessions, tailored to individual cultural or religious beliefs. These methods, amongst others, have been shown to improve healthcare literacy in those population groups from the most deprived regions, and may also be influential in improving understanding and subsequent engagement with diabetes technology [34,35]. Targeted education of the healthcare professionals themselves is equally impactful in ensuring that those populations from the most deprived quintiles have improved access and opportunity to engage with diabetes technology, therefore improving healthcare equality across all deprivation quintiles [36].

Limitations

Some limitations to this study were identified. As this was an observational, single-centre study conducted in the Mid and South Essex NHS Foundation Trust, due to the unique demographics and infrastructure of each NHS Trust locality, it is possible that the collected data and subsequent inferences could be skewed and therefore inappropriate for application to other regions within the UK [37,38]. As such, the inclusion of different regions exhibiting different degrees of disparities is indicated in order to construct a multi-centre analysis that will be more useful in drawing NHS-wide conclusions [39]. The impact of ethnicity and deprivation on access to diabetes technology has been highlighted in the UNBIASED study, which included multiple NHS trusts, demonstrating that young people from less deprived socioeconomic backgrounds benefit more from diabetes technologies [40]. Notably, the study was not limited to paediatric patients; however, it did indicate the impact of multi-centre studies in providing more valid data in investigating this important subject.

As a retrospective observational study, causal inferences cannot be drawn. Although we observed relationships between socioeconomic deprivation, access to diabetes technology, and glycaemic outcomes, we cannot determine the directionality or whether other unmeasured factors may explain these associations. Although our findings suggest that access to insulin pump therapy may partially mediate the relationship between socioeconomic deprivation and glycaemic outcomes, no formal mediation analysis was performed. Further studies with appropriate adjustment for confounders and a longitudinal design are required in order to assess this hypothesis.

Some selection bias is also identified regarding those patients ‘awaiting’ insulin pump therapy, as this includes those patients who have agreed to the use of insulin pump therapy in clinic or have discussed the matter at a minimum [41]. Systematically, these patients may be skewed towards the higher quintiles, as they are more likely to be engaged with the care and follow-up of this chronic disease, or may have better support systems in place. As such, this may not be an accurate data point representative of the population. 

Of the 48 pump users, only 35 had complete pre- and post-initiation HbA1c data, thereby limiting the required included population to reach sufficient statistical power to elicit statistically significant interventions. Involvement from more centres would be needed to achieve this required population. As HbA1c data were missing for a proportion of pump users, the analysis was limited to patients with complete pre- and post-initiation HbA1c values. This may have introduced bias, as those with missing data may differ systematically from those with complete data. Finally, regarding the logistical regression approximation performed, there are confounders that were not adjusted for, such as those factors that may limit exposure and engagement with technology, including language barriers and psycho-social factors [42]. These confounders include diabetes duration, parental engagement, and clinic attendance, which were not available for inclusion. We acknowledge that these unmeasured confounders may influence both access to technology and glycaemic outcomes.

## Conclusions

This study highlights the effects of socioeconomic deprivation on technology use in CYD with T1DM, with CYD in the least deprived quintile and those of White ethnicity having greater access to diabetes technology. CYD across all deprivation quintiles experienced improved glycaemic control with technology use, suggesting improving access to technology may reduce glycaemic disparities in the more deprived populations. While glycaemic outcomes improved with insulin pump use across all quintiles, the findings should be interpreted cautiously due to the observational design of this study and potential for unmeasured confounding. Strict health board criteria for diabetic technology approval may contribute to reduced participation among the most deprived quintiles due to lack of awareness and exposure. Further research addressing organisational and individual factors affecting these health disparities is required.
